# Prospective observational study of FKRP‐related limb‐girdle muscular dystrophy R9: A GRASP consortium study

**DOI:** 10.1002/acn3.52276

**Published:** 2024-12-15

**Authors:** Lindsay N. Alfano, Meredith K. James, Kristine Grosfjeld Petersen, Karen Rudolf, John Vissing, Renee Augsburger, Tahseen Mozaffar, Aileen Jones, Amanda Butler, Katie M. Laubscher, Shelley R. H. Mockler, Katherine D. Mathews, Megan A. Iammarino, Natalie F. Reash, Lindsay Pietruszewski, Linda P. Lowes, Talia Strahler, Matthew Wicklund, Stephanie Hunn, Conrad C. Weihl, Sandhya Sasidharan, Melissa Currence, Jeffrey M. Statland, Nikia Stinson, Megan Holzer, Doris G. Leung, Donovan J. Lott, Peter B. Kang, Scott Holsten, Urvi Desai, Nicholas E. Johnson, the GRASP‐LGMD Consortium

**Affiliations:** ^1^ Center for Biobehavioral Health The Abigail Wexner Research Institute at Nationwide Children's Hospital Columbus Ohio USA; ^2^ Department of Pediatrics The Ohio State University College of Medicine Columbus Ohio USA; ^3^ The John Walton Muscular Dystrophy Research Centre Newcastle upon Tyne Hospitals NHS Trust and Newcastle University Newcastle Upon Tyne UK; ^4^ Department of Neurology, Copenhagen Neuromuscular Center, Rigshospitalet University of Copenhagen Copenhagen Denmark; ^5^ Department of Neurology University of California Irvine California USA; ^6^ Department of Neurology Virginia Commonwealth University Richmond Virginia USA; ^7^ Center for Disabilities and Development University of Iowa Health Care Stead Family Children's Hospital Iowa City Iowa USA; ^8^ Department of Neurology University of Iowa Iowa City Iowa USA; ^9^ Department of Neurology University of Colorado School of Medicine Aurora Colorado USA; ^10^ Department of Neurology Washington University School of Medicine St. Louis Missouri USA; ^11^ Department of Neurology University of Kansas Medical Center Kansas City Kansas USA; ^12^ Center for Genetic Muscle Disorders Kennedy Krieger Institute Baltimore Maryland USA; ^13^ Department of Neurology Johns Hopkins Medicine Baltimore Maryland USA; ^14^ Department of Physical Therapy University of Florida Gainesville Florida USA; ^15^ Department of Pediatrics University of Florida Gainesville Florida USA; ^16^ Department of Neurology Atrium Health Charlotte North Carolina USA; ^17^ Present address: Department of Neurology, and Institute of Translational Neuroscience, Greg Marzolf Jr. Muscular Dystrophy Center University of Minnesota Minneapolis Minnesota USA

## Abstract

**Objective:**

Limb‐girdle muscular dystrophy R9 (LGMDR9, formerly known as LGMD2I), caused by variants in the fukutin‐related protein (FKRP) gene leads to progressive muscle weakness of the shoulder and pelvic limb‐girdles and loss of motor function over time. Clinical management and future trial design are improved by determining which standardized clinical outcome assessments (COA) of function are most appropriate to capture disease presentation and progression, informing endpoint selection and enrollment criteria. The purpose of our study was to evaluate the cross‐sectional validity and reliability of clinical outcome assessments in patients with FKRP‐related LGMDR9 participating in the Genetic Resolution and Assessments Solving Phenotypes in LGMD (GRASP) natural history study.

**Methods:**

Enrolled patients completed a battery of COA on two consecutive days, including the North Star Assessment for limb girdle‐type dystrophies (NSAD), the 100‐m timed test (100 m), and the Performance of Upper Limb 2.0 (PUL).

**Results:**

A total of 101 patients with FKRP‐related LGMDR9 completed COA evaluations. All functional COA were highly and significantly correlated even across constructs, except for the 9‐hole peg test. Similarly, all tests demonstrated excellent test–retest reliability across 2‐day visits. The NSAD and PUL demonstrate robust psychometrics with good targeting, ordered response thresholds, fit and stability, and limited dependency of items across the scales.

**Conclusions:**

This study has determined the suitability of several functional COA, cross‐sectionally, in LGMDR9 to inform future trial design and clinical care.

## Introduction

Limb‐girdle muscular dystrophies (LGMD) are rare, progressive, and clinically heterogenous neuromuscular disorders. Variants in the fukutin‐related protein (FKRP) gene can result in a range of phenotypes from childhood‐onset Walker‐Warburg syndrome/muscle‐eye‐brain disease to adult‐onset FKRP‐related LGMDR9.^1^, ^2^, ^3^ FKRP contributes to the glycosylation of α‐dystroglycan, a part of the dystrophin‐glycoprotein complex, which anchors muscle fibers to the extracellular matrix.^4^ Decreased glycosylation of FKRP impacts repair of muscle contraction‐induced injury as well as signal transduction from the extracellular matrix.

LGMDR9 is a common form of LGMD globally, though prevalence varies regionally due to the influence of a Northern European founder mutation.^5^, ^6^, ^7^ Many patients with LGMDR9 have a homozygous founder variant (c.826C>A) in the *FKRP* gene, and natural history studies suggest that patients with compound heterozygous variants present at younger ages and experience a more significant decline in childhood compared to those with the homozygous founder variant.^8^, ^9^ Muscle weakness or hyperCKemia are the most common presenting signs or symptoms. Time to diagnosis, although improving, has been reported to take a median of 6.5 years; however, it can take longer for those with acute or intermittent presenting symptoms.^10^ The prevalence of cardiomyopathy varies by reported cohort but ranges between 23 and 55% of patients with median age of onset around 50 years of age for homozygotes and late teens to 20 years for heterozygotes.^9^, ^11^, ^12^ Noninvasive respiratory support occurs at around 40 years of age in homozygotes and varies widely in compound heterozygotes; the median age at initiation ranges between 13 and 39 years and can precede loss of ambulation in some patients.^5^, ^9^ Similarly, insomnia and sleep‐disordered breathing are likely underrecognized.^13^, ^14^ Pain is commonly reported in patients with LGMDR9 and interferes with daily life in a majority of patients studied through a global FKRP patient registry.^15^ Fatigue occurs in adulthood, increases with age, and is related to motor and pulmonary function.^15^ However, a paradoxical decline in disease burden and fatigue have also been reported in patients with LGMDR9 who are full‐time wheelchair users compared to those using walking aids.^14^

While there are no current disease‐modifying treatments approved for use in LGMDR9, various approaches from small molecules to gene therapy are being developed and investigated.^16^, ^17^, ^18^, ^19^, ^20^, ^21^, ^22^ Prospective observational studies enable data‐driven study design by informing study enrollment criteria, selection of appropriate safety and efficacy outcomes, as well as interpretation of trial results. While several groups have contributed to our general understanding of the LGMDR9 phenotype, there remains a dearth of validated outcomes or prospective validation of outcomes in patients with LGMDR9. The Genetic Resolution and Assessments Solving Phenotypes in LGMD (GRASP‐LGMD) consortium was established in 2019 to validate clinical outcomes across various LGMD subtypes, including LGMDR9, and includes sites in the United States and Europe.

The primary objective of this study was to evaluate the cross‐sectional validity and reliability of clinical outcome assessments in patients with LGMDR9. Additionally, investigators sought to evaluate the psychometric properties and underlying construct of the North Star Assessment for limb girdle‐type dystrophies (NSAD) and Performance of Upper Limb (PUL) via Rasch‐based methodology.

## Materials and Methods

Institutional review board approval was obtained for the study protocol and oversight was provided for all study activities. Participants provided written informed consent and/or assent, when appropriate. Details of the study were posted on clinicaltrials.gov (NCT04202627).

Participants between the ages of 10 and 65 years (inclusive) with two pathogenic variants or one pathogenic with one variant of unknown significance in the *FKRP* gene were enrolled. Participants were required to be ‘clinically affected’ by LGMDR9, defined as weakness on bedside evaluation in either a limb‐girdle or distal extremity presentation. Those with a 10‐m walk/run time (10 m) of <4 sec; positive pregnancy test; history of a bleeding disorder; known platelet count <50,000 or current use of an anticoagulant; or with any other comorbidity that would impact the safety of participation in this study were excluded.

Eligible participants were enrolled in a 12‐month study with visits as baseline, 6, 9, and 12 months. At each study visit, participants performed a battery of testing in a standardized order including the 100‐m timed test (100 m), NSAD that includes timed rise from floor (RFF) and 10‐m walk/run (10 m), 4 stair climb (4SC), Timed up and go (TUG), PUL, and 9‐hole peg test (9HPT).^23^, ^24^, ^25^, ^26^, ^27^, ^28^, ^29^ Here we report the cross‐sectional analysis of the enrollment cohort at the 2‐day baseline visits.

The cohort was divided into two groups by 10 m time performance at baseline Day 1 visit: Group A: 10 m ≤12 sec, Group B: unable to walk 10 m safely or 10 m >12 sec. Participants enrolled in Group A performed all functional assessments, but those in Group B completed assessments focused on upper extremity function (i.e., NSAD, PUL, and 9HPT) to reduce the burden of testing and focus on the most relevant and meaningful functional measures. All strength and functional COA were administered in a standardized order and repeated on two consecutive days to establish test–retest reliability.

Similarly, participants completed several patient‐reported outcomes (PRO) including the ACTIVLIM; Disabilities of the arm, shoulder, and hand (DASH); Patient‐reported outcomes measurement information system (PROMIS)‐57; and the Limb Girdle Muscular Dystrophy Health Index (LGMD‐HI).^30^, ^31^, ^32^, ^33^, ^34^, ^35^ Participants, or their parent/guardian for children under 18 years of age, completed the PRO once across the 2‐day baseline visit.

### Statistical analysis and psychometric evaluation

Data analysis was performed with SPSS software version 29 (IBM SPSS, Chicago, IL, USA). Descriptive statistics were used to quantify participant demographics. General linear regression modeling was used to evaluate the impact of variant group and time since reported symptom onset on performance of clinical outcome assessments (COA). Test–retest reliability of NSAD scores was assessed using intraclass correlation coefficient (ICC2, 1) and Bland–Altman plots. Independent *t*‐tests were used to evaluate the effect of age, variant group, age at symptom onset, age at diagnosis, and current ambulatory status. Spearman correlation coefficients were used to explore the relationship between COA and PRO.

### Psychometric evaluation using Rasch unidimensional measurement model (RMM)

The psychometric performance of NSAD and PUL was completed using RMM and examined across seven areas: targeting, response categories, fit, reliability, dependency, stability, and unidimensionality.^36^ Available baseline Day 1and Day 2 data were entered into RUMM 2030 software.^37^

### Targeting and response categories

Targeting investigates the match between items of scale and the range of functional ability of the cohort. A robustly designed scale should contain items that cover the range of expected abilities within the tested patient population. Response categories evaluate individual item scoring to ensure that they reflect an ordered continuum of disease progression or potentially better function with an intervention. Ordered scoring thresholds demonstrate scale validity. Threshold locations and plots are utilized by RMM to examine response categories statistically and graphically.^38^

### Fit

Evaluation of fit ensures items fit statistically and clinically, otherwise, a summed total score may be inappropriate. All items should lie within a fit residual standard deviation (SD) range of ±2.5 to be considered to represent an appropriate fit to the measured construct. If the person and item fit the model are good, the z‐score mean is around 0 and an SD of 1 is expected. The third fit statistic is the item‐trait interaction statistic, reported as a chi‐squared (*χ* ^2^) value, where a significant chi‐squared value indicates an item misfit to the total scale.

### Reliability

Scale reliability was quantified using the Person Separation Index (PSI) (similar to the Cronbach alpha), which compares the observed variance to true scale variance.^39^, ^40^ Greater reliability is indicated with a higher PSI.

### Dependency

Dependency estimates evaluate if responses to items disproportionately impact the response to another item (i.e., items have highly correlated residuals), as this may bias measurement estimates and cause artificial elevation of reliability by PSI.^41^, ^42^

### Stability and unidimensionality

The stability of the scale's item performance was determined by examining differential item functioning (DIF) across subgroups‐ age, sex, and genetic variant (homozygote vs heterozygote). As the cohort ranges in age between 10 and 64 years, two age categories were used, adult (>18 years) and child (10–18 years). The fundamental requirement for RMM is that the items of the scale measure a single construct, the presence of unidimensionality was determined using principal components analysis and reported with a *t*‐test.

## Results

One hundred and one patients were enrolled in the study, with demographics found in Table 1. A majority of the cohort were ambulatory and included in Group A at baseline (*n* = 68) as expected per a protocol restriction on the enrollment size of Group B. Seventy‐three percent of the total cohort were homozygous for the c.826C>A (p.Leu276Ile) founder variant. There were no significant differences in age, age at diagnosis, or age at symptom onset between Groups A and B at enrollment. As expected, there was a significant difference in age at symptom onset between genotype groups, with compound heterozygotes exhibiting an earlier symptom onset than the homozygote group. Sixteen patients reported the use of ribose and three reported using corticosteroids for muscle weakness related to LGMD (two with deflazacort, one with prednisone) at baseline. Ten participants were treated with spironolactone (*n* = 9) or eplerenone (*n* = 1) related to cardiac care. Four additional patients reported prior exposure to steroids to treat muscle weakness associated with LGMD. There was no significant difference in performance on functional outcomes in those with or without exposure to these treatments in our small sample (*P* > 0.05). Twelve patients completed their baseline visits remotely due to the COVID‐19 pandemic travel‐related restrictions.

**Table 1:** Cohort demographics at enrollment with subgroupings by ambulatory status and allele classification.

	*N*	Mean ± SD	Range	*P*
Age	101	36.2 ± 14.6	10–64	
Ambulatory	68	33.5 ± 14.4	10–64	ns
Nonambulatory	33	41.7 ± 13.6	15–62	
Heterozygous	27	28.3 ± 14.4	10–58	ns
Homozygous	74	39.1 ± 13.6	10–64	
Pediatric	16	13.9 ± 2.3	10–17	<0.001
Adult	85	40.5 ± 11.5	21–64	
Symptom onset	101	15.9 ± 11.1	0–50	
Ambulatory	68	17.1 ± 10.9	0–50	ns
Nonambulatory	33	13.6 ± 11.1	0–38	
Heterozygous	27	9.3 ± 7.4	0–26	0.02
Homozygous	74	18.4 ± 11.2	0–50	
Pediatric	16	5.1 ± 4.2	0–14	0.001
Adult	85	17.9 ± 10.8	0–50	
Diagnosis age	101	25.8 ± 14.4	1–60	
Ambulatory	68	25.6 ± 14.1	2–59	ns
Nonambulatory	33	13.6 ± 11.1	1–38	
Heterozygous	27	15.6 ± 12.5	1–51	ns
Homozygous	74	30.0 ± 13.2	5–60	
Pediatric	16	7.8 ± 4.4	1–16	<0.001
Adult	85	29.2 ± 12.9	2–60	

### Cross‐sectional performance of COA and PRO

**Figure 1:**
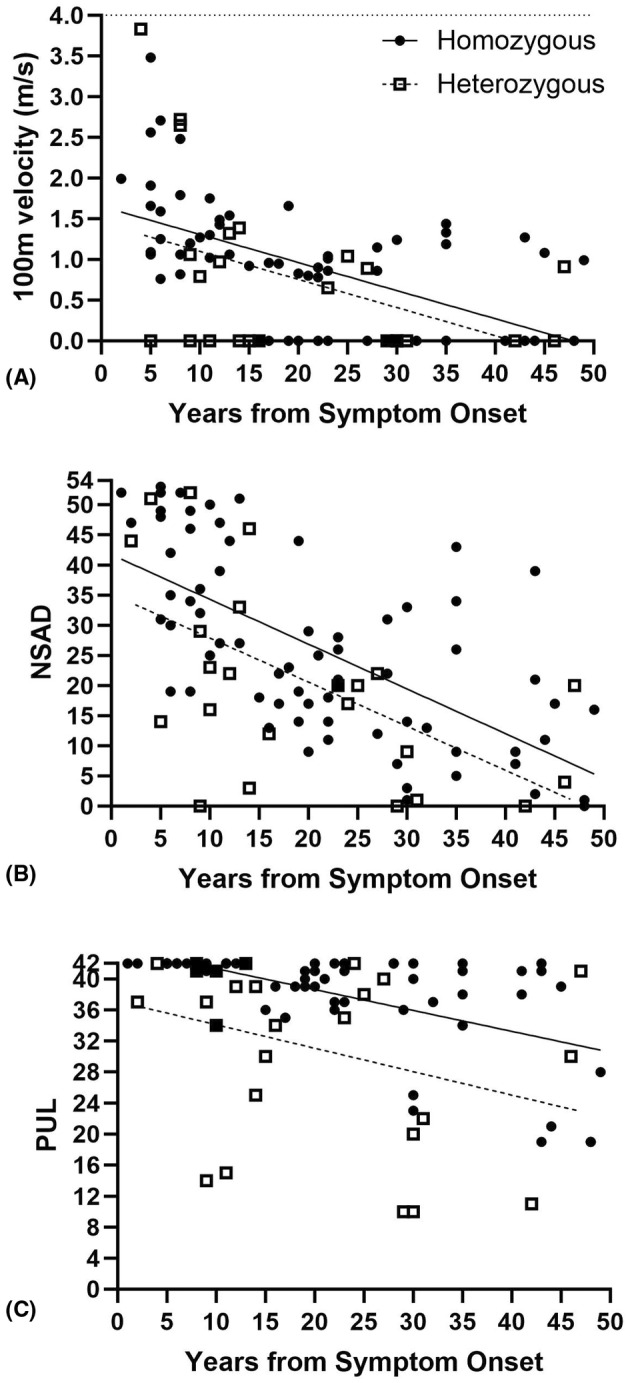
Performance of key clinical outcome assessments (COA) plotted by years since report of symptom onset (A) 100‐m timed test (100 m) converted to velocity, (B) North Star Assessment for limb girdle‐type dystrophies (NSAD), and (C) Performance of Upper Limb (PUL). Note patients are plotted by variant group (circles = homozygous, squares = compound heterozygous) and linear regression lines (solid = homozygous, dotted = compound heterozygous) indicate differences in age of onset but similar slope of progression over time.

See Table 2 for descriptive statistics of each COA across the cohort including the total number of patients with valid results at baseline and average scores or times with range of performance on Day 1 and Day 2. A valid time was recorded for the 100 m in 56 patients as they traversed the full 100 m without an assistive device or external assistance. One third of the cohort was nonambulatory at baseline, seven patients were unable to record a valid time due to space constraints within the home environment during their remote baseline visit, and an additional five patients had missing data. Converting values to a velocity provides a valid result for 89 patients across the cohort with those unable to complete the test due to functional limitations receiving a value of 0 m/s (*N* = 33). Figure 1A demonstrates the performance of this COA by time since reported symptom onset and separated by variant group. Patients with compound heterozygous variants exhibited an earlier onset of weakness than homozygotes. No patients in our cohort achieved a running speed of 4.0 m/s or greater, indicating limited or no ceiling effect of this COA.^23^, ^43^, ^44^ Twenty‐seven patients walked at or above 1.2 m/s considered the minimum threshold for community ambulation speed; five patients were transitionally ambulatory and walking at or below 0.8 m/s classifying them as functionally ambulatory within a household environment only.^44^

**Table 2:** Group average performance ± standard deviation and range of performance at baseline Day 1 and Day 2 on each functional clinical outcome assessment (COA), average group change ± standard deviation and range, and test–retest reliability (intraclass correlation coefficient).

COA	*N*	Day 1	Day 2	Change	ICC*
100 m (s)	56	84.6 ± 29.1 26.1–154.0	85.2 ± 28.9 28.9–148.0	−0.2 to 4.3 −15.7 to 9.3	0.99
NSAD	94	25 ± 16 0–53	25 ± 15 0–53	0.1 ± 1.4 −7 to 4	0.99
RFF (s)	29	6.6 ± 5.5 1.0–23.7	8.0 ± 6.5 1.2–25.0	−0.8 ± 1.6 −5.5 to 1.4	0.96
10 m (s)	68	7.7 ± 4.2 2.2–28.2	8.0 ± 3.7 2.2–24.4	−0.1 ± 1.3 −7.5 to 3.8	0.95
4SC (s)	53	6.7 ± 5.2 1.3–27.2	6.7 ± 5.6 1.3–30.8	0.0 ± 0.9 −3.6 to 1.8	0.99
TUG (s)	57	10.7 ± 5.8 3.2–32.4	10.9 ± 5.9 3.1–34.8	−0.4 ± 2.2 −13.8 to 3.3	0.92
PUL	94	36 ± 8 10–42	36 ± 8 10–42	0.1 ± 0.8 −3 to 3	0.99
9HPT (s)	66	25.5 ± 23.3 14.2–172.0	26.0 ± 36.9 13.6–300.0	−0.8 ± 16.5 −128.0 to 11.6	0.86

A negative change in time is indicative of improved performance as the time decreased across days.

10 m, 10‐m walk/run; 100 m; 100‐m timed test; 4SC, 4 stair climb; 9HPT, 9 hole peg test; COA, clinical outcome assessments; ICC, intraclass correlation coefficient; *N*, number of participants with valid data across days; NSAD, North Star Assessment for limb‐girdle type dystrophies; PUL, Performance of Upper Limb; RFF, rise from floor; TUG, timed up and go.

* *P* < 0.001 for all COAs.

Baseline NSAD data exist for 94 patients with four patients scoring 0 and seven additional patients scoring ≤4 points, indicating a slight floor effect of this assessment in a nonambulatory cohort. Six of these patients had compound heterozygous variants with time since symptom onset ranging 9–46 years; the remaining five had homozygous variants with 30–48 years since reported symptom onset. No patient received a full score on NSAD at baseline. Figure 1B demonstrates the performance of NSAD across the cohort separated by variant group, with patients with compound heterozygous variants exhibiting an earlier onset of weakness than homozygotes.

Short‐timed functional tests included in our battery were rise from floor (RFF) and the 10‐m walk/run (10 m), both included in the NSAD, as well as the 4‐stair climb (4SC) and Timed Up and Go (TUG). RFF was informative in <20% of our cohort as only 29 patients could complete the test without the use of furniture but 10 of those completed the test in <3 sec, which is indicative of typical performance. While the clinical relevance of this COA is clear, this ability is quickly lost once weakness presents rendering it less useful as a potential clinical trial outcome. Walking or running velocity for the 10 m velocity was available for 97 patients as those unable to complete the test safely without assistive devices (*n* = 18) were recorded as 0 m/s to be included in the analysis. Interestingly, patients taking ≥7 sec to complete the 10 m were likely to have lost the ability to RFF independently as only 6 of 12 (50%), 2 of 7 (29%), and 2 of 12 patients (17%) completing the 10 m in 7, 8, or 9 sec, respectively, could RFF without furniture. Similarly, all patients taking >10 sec to complete 10 m were unable to RFF without furniture or assistance. The 4SC could be completed in 60 patients (53 with valid times, 7 additional were able but no time was recorded due to remote testing at baseline visit). Sixteen patients completed the test in <3 sec, indicative of typical performance, thus the 4SC would be informative in just 46% of the total cohort without floor or ceiling effects impacting interpretability. TUG was successfully performed in 91 patients at baseline (57 patients completed and 34 were unable). Of those completing the TUG, 37% took longer than 11 sec (range 11.9–32.4 sec) putting them at a high risk for falls.^45^, ^46^, ^47^, ^48^ Remote testing did not impact the ability to capture this assessment as a 3‐m walking space and a stable chair were available within all home environments.

Upper extremity testing included the PUL and the 9‐hole peg test (9HPT). A valid PUL score was available for 94 patients at baseline with 34 of those (36%) patients achieving the maximum score of 42, including three non‐ambulant individuals with a homozygous variant, indicating a ceiling effect of this assessment in LGMDR9. Figure 1C demonstrates the variable time to upper extremity involvement across the cohort, with patients with compound heterozygous variants exhibiting an earlier onset of upper extremity weakness than homozygotes and progressing slightly more rapidly (*P* < 0.001). Similarly, while nonambulatory patients are generally expected to have more pronounced upper extremity involvement, 20% of ambulatory participants demonstrated mild to moderate upper extremity weakness ranging between 2 and 18 years from diagnosis, and between 12 and 55 years of age; further supporting the heterogeneity of muscle involvement and progression of disease in patients with LGMDR9. The lowest recorded score at baseline was 10 points indicating no floor effect of PUL in our cohort. Most patients could at least raise their hands to mouth, with only seven patients, all nonambulatory (age range 16–55 years), confined to tabletop activities only. Items falling in the distal dimension begin to be more challenging for patients with PUL Entry item ≤3, but a larger spread of scores was observed for those with an Entry item score of 1. When completing the 9HPT, only 9 patients took longer to complete the test than would be expected for their age and sex, indicating there is a significant ceiling effect for this COA in this cohort.^49^, ^50^ This COA is likely most informative in a cohort of patients with significant upper extremity involvement, scoring “1” on the PUL entry item.

The various PRO included in this study had similar usefulness or limitations across our cohort. It is important to note that our surveys were available in English, which reduced their implementation for patients fluent in other languages. When responding to ACTIVLIM items (higher score indicative of greater abilities), five patients scored within two points of the maximum score at baseline (34–36 points), and three nonambulatory patients scored less than five points indicating significant activity limitations and a potential floor effect in patients with LGMDR9. The most challenging items across all patients were “Having a bath” and “Walking upstairs.” Adults ranked “Carrying a heavy load” and “Walking more than 1 km” as the most challenging. “Closing a door” was reported as easiest for children, and “Washing one's face” was scored least challenging across all patients. Two patients had a ceiling effect when reporting abilities using the DASH (lower score indicative of greater abilities); both also experienced a ceiling effect on the ACTIVLIM. The highest score, indicative of lower abilities, was 70.8 out of 100 possible points on the DASH, indicating no floor effect of this assessment in this cohort. Sixty patients reported working at baseline, but only 21 were involved in sports activities. Patients with employment at baseline reported mild to moderate difficulty performing work tasks, while those playing sports or an instrument reported moderate to severe difficulty on average. When evaluating the LGMD‐HI, six patients achieved ceiling effect when summing reports across the Physical Health domains of the scale. The items most significantly and meaningfully impacting a majority of the cohort were related to leg weakness and mobility.

The PROMIS‐57 measures patient‐reported function and quality of life measures across seven domains in total. Here we report on the mobility‐related domains of physical health, fatigue, social participation, and pain. When evaluating the physical health domain, five patients demonstrated a ceiling effect which correlated with the same and strongest patients on the other PRO. These patients were below 18 years of age apart from one patient who was 47 years (homozygous variant group) at baseline. In addition, 10 patients scored <10 points demonstrating a floor effect. PROMIS‐57 also includes the ability to convert raw scores to normative T‐scores. At baseline 42 patients scored >1 SD below average expectations for physical health and 18 scored >2 SD below. Just 11 patients reported very minimal to no impact of fatigue on their daily lives at baseline. However, nine patients reported fatigue at a level reaching >1 SD more fatigue than expected for age, and two patients >2 SD. Most patients (80%) reported some impact of LGMDR9 on their social participation, though 10 patients reported no issues. Four patients demonstrated severe impact and reached a floor effect on this portion of the PRO. In our sample, 20 patients demonstrated reduced social participation reaching >1 SD below expectation for age. Last, pain was reported across the cohort with all patients reporting at least mild impact of pain on daily life with an average intensity of 2.7 ± 2.1 (on a scale from 0 = no pain to 10 = worst pain imaginable). Severe pain was reported in 33 patients, with these patients also reaching a floor effect on this PRO.

### Relationship between COA and PRO

**Figure 2:**
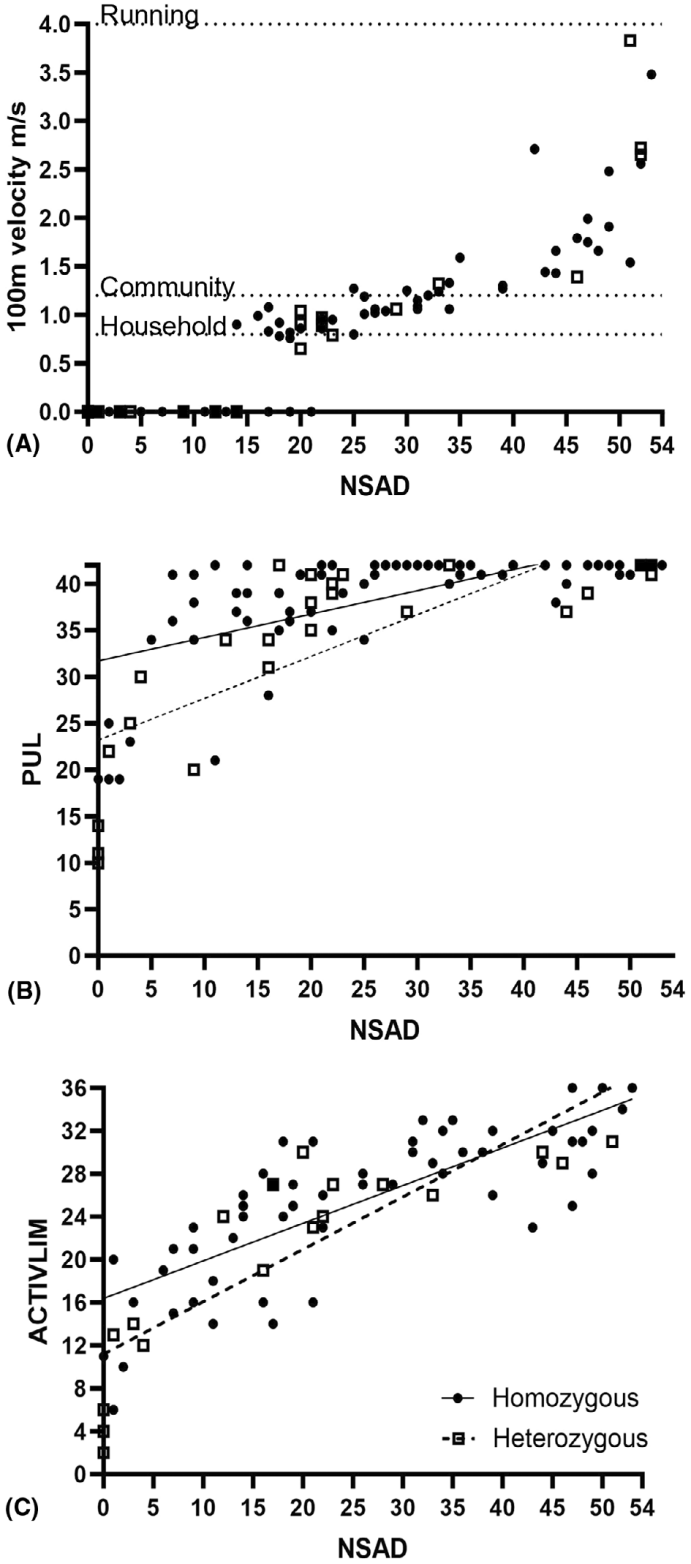
Relationship between key COA (A) NSAD and 100 m. No patients reached top running velocity with a good distribution of abilities over the course of disease progression. Thresholds for typical “running,” “community” ambulation, and “household” ambulation are marked as dotted lines. (B) NSAD and PUL indicate a greater spread of NSAD score over time with PUL increasingly informative as ambulatory ability declines. (C) NSAD and ACTIVLIM demonstrate increasing patient‐reported difficulty with activities correlating to clinician‐measured function. Note the mild floor effect of the ACTIVLIM as patients become nonambulatory. Patients with homozygous variants are marked as black circles (solid line) and patients with compound heterozygous variants are marked as open squares (dashed line).

All ambulatory assessments correlated significantly (*r* ≥ 0.66, range: 0.66–0.90; *P* < 0.001). The PUL significantly correlated most highly with the NSAD (*r* = 0.67, *P* < 0.001), 100 m (*r* = −0.44, *P* < 0.001), and the ACTIVLIM (*r* = 0.90, *P* < 0.001). The 9HPT was significantly related to the PUL (*r* = −0.58, *P* < 0.001), but no other functional COA was measured. Figure 2C demonstrates the relationship between patient‐reported activity, measured by the ACTIVLIM, and clinician‐observed functional abilities, measured by the NSAD (*r* = 0.88, *P* < 0.001) (Table 3).

**Table 3:** Spearman's correlation coefficient comparing patient‐reported function to performance on clinic‐based functional assessments.

PRO	NSAD	10 m	100 m	TUG	PUL
ACTIVLIM	0.88^†^	−0.54*	−0.58*	−0.57*	0.85^†^
PROMIS‐57: physical health	0.82^†^	−0.51^†^	−0.61^†^	−0.63^†^	0.63^†^
DASH	−0.72^†^	0.43*	0.54^†^	0.55^†^	−0.64^†^
LGMD‐HI: physical function	−0.50^†^	0.49**	0.56^†^	0.55^†^	−0.34*

10 m, 10‐m walk/run; 100 m; 100‐m timed test; DASH, disabilities of the arm, shoulder, and hand; LGMD‐HI; the Limb Girdle Muscular Dystrophy Health Index; NSAD, North Star Assessment for limb‐girdle type dystrophies; PRO, patient‐reported outcomes; PROMIS, patient‐reported outcomes measurement information system; PUL, Performance of Upper Limb; TUG, timed up and go.

* *P <* 0.05.

** *P <* 0.01.

^†^ *P <* 0.001.

Correlation coefficients comparing patient‐reported ability in functional domains of each included PRO to their performance on functional COA are listed in Table 3. Of note, ACTIVLIM correlates most highly across outcomes, followed by the physical health domain of PROMIS‐57.

### Test–retest reliability

Group‐level performance of functional COA was consistent between baseline Day 1 and Day 2 with ICC indicating excellent test–retest reliability (Table 2). The 9HPT demonstrated the most variability between days.

**Figure 3:**
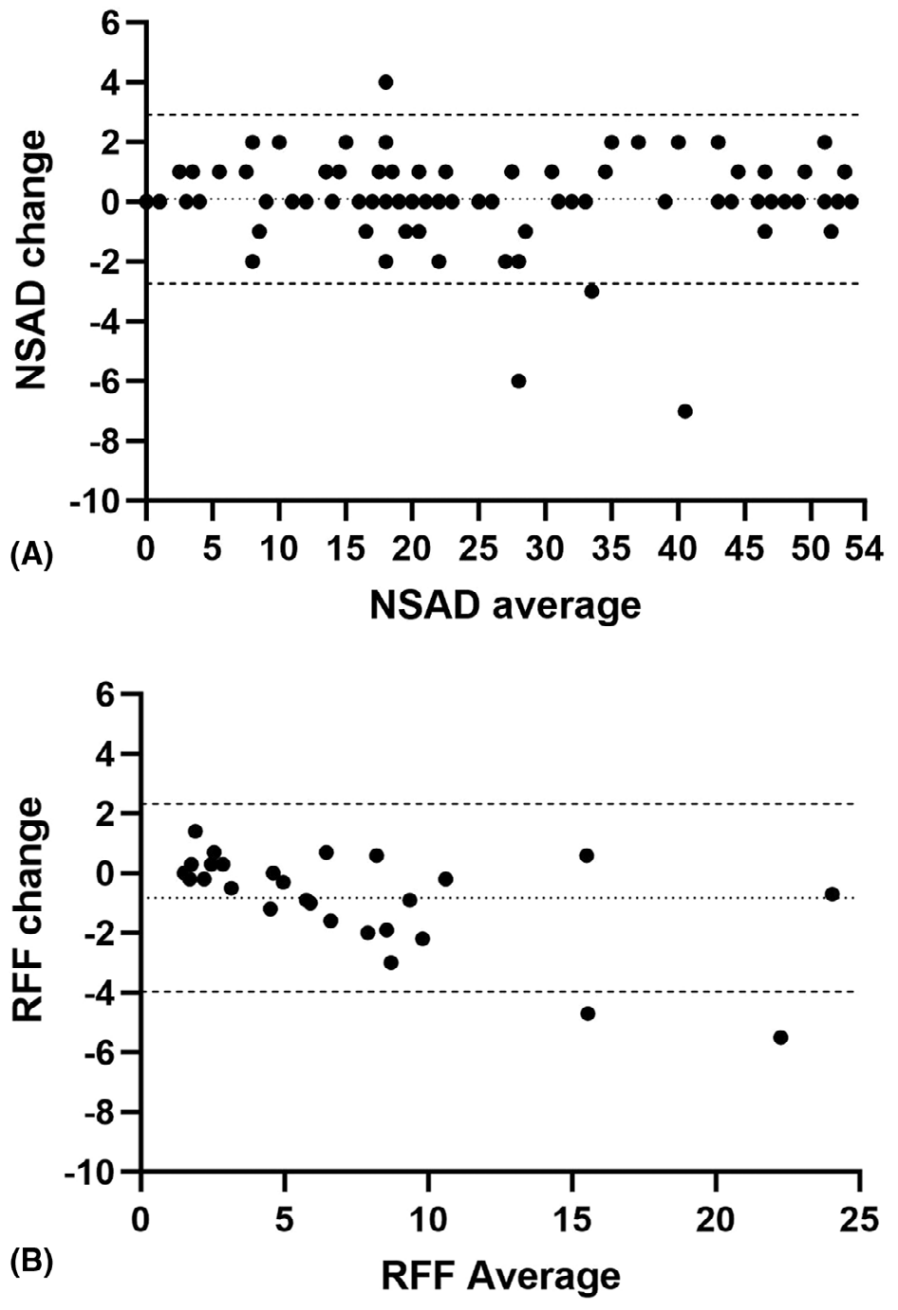
Bland–Altman plots of performance of functional outcomes across days which includes individual average performance (Day 1 and Day 2) and change across baseline visits for (A) North Star Assessment for limb girdle‐type dystrophies (NSAD), (B) rise from floor (RFF). Note data for RFF were transposed to indicate decreased performance (meaning increasing time) across days are displayed as a negative number for ease of interpretation and comparison to NSAD as an ordinal variable with decreasing score indicative of worsening performance.

While group‐level performance is consistent across days, it is important to note some individual patient variability exists. Bland–Altman plots demonstrate what appears to be random variability in a few individual patients on stable assessments like the NSAD and PUL (Fig. 3A), or trends of decline across visits which may be attributed to fatigue across days (Fig. 3B). The range of change in individual patient scores is listed in Table 2.

### Psychometric evaluation of NSAD and PUL using RMM

Available data from 189 NSAD and 192 PUL assessments were entered into RUMM2030 software. Summary findings in Table 4. Both the NSAD and PUL demonstrated unidimensional constructs and high reliability with a PSI of 0.97 and 0.86 respectively. Two distal items, pick up 6 tokens and pick up a 10‐gram weight were removed from the analysis as all patients received a full score on these items and they were redundant in this primarily ambulant cohort.

**Table 4:** NSAD and PUL psychometric evaluation summary using Rasch mathematical methods.

Scale	Item fit	Person fit	Reliability	Item fit		Dependency	Unidimension‐ality
	Mean (SD)	Mean (SD)	PSI with extremes	Ordered thresholds	Number of items with good fit	Number of pairs	
NSAD	−0.42 (1.83)	−0.26 (0.68)	0.97	28/29 (97%)	27/29^a^ 5 significant *χ* ^2^ ^b^	13 pairs >0.3 (PSI 0.96 with one of each pair removed)	Acceptable (*t*‐test 0.038, 95% CI 0.006–0.069)
PUL	−1.58 (2.00)	−0.20 (0.45)	0.86	19/20 (95%)	15/20^a^ 5 significant *χ* ^2^ ^b^	16 pairs >0.3 (PSI 0.86 with one of each pair removed)	Acceptable (*t*‐test 0.046, 95% CI 0.016–0.076)

^a^ Fit residual inside the recommended range (−2.50 to 2.50) and ^b^significant *χ* ^2^ probability (*P* < 0.01).

NSAD, North Star Assessment for limb‐girdle type dystrophies; PUL, Performance of Upper Limb.

### Targeting

The NSAD items targeted both ambulant and weaker non‐ambulant cohorts, without any significant ceiling or floor effects (Fig. S1). Item locations spread from 4.2 to −4.5 indicating a good continuum of coverage with little overlap. A mild floor existed for the very weakest patients with no independent pelvic or shoulder girdle movement. Overall item‐trait interaction *χ* ^2^ value was 296.71 (58 df).

The PUL items targeted those with involvement of the upper limb and included items that all the weakest of the cohort were able to perform (Fig. S3). A ceiling exists for patients without involvement of the shoulder girdle. Item locations from −5.351 to 4.339 with a good spread of items. Overall item–trait interaction *χ* ^2^ value was 353.48 (40 df).

### Response categories

*NSAD*: 28 of 29 items demonstrated ordered response categories (Fig. S2), with the remaining item (rolling) approaching an ordered threshold and will remain with that scoring.

*PUL*: 19 of 20 items with ordered response categories (Fig. S4), with only the shoulder abduction to shoulder height item approaching ordered thresholds.

### Fit

When evaluating the NSAD, two items had a misfit, four with *χ* ^2^ *P* < 0.01 (Table S1). The misfitting items were sit‐to‐stand and stand‐on tiptoes. In the context of other parameters, these two items functioned well, have significant clinical meaning, and were retained in the scale. The PUL had five items with misfit, and 5 with *χ* ^2^ *P* < 0.01 (Table S2). The misfitting items were shoulder flexion to shoulder height, shoulder flexion above shoulder height with 500 g, and supination.

### Dependency

The NSAD exhibited 13 pairs of items with highly correlated residuals (>0.3) (Table S1). These pairs were items assessing bilateral performance or component parts of the same movement (i.e., squatting down and standing from squat, standing on the right or left leg). With one item from each pair of dependent items removed, the PSI was re‐calculated. The PSI remained stable at 0.96, indicating the dependency had not artificially inflated the PSI. Items that test the ability on the left and right sides of the body were considered clinically meaningful, with improved sensitivity to asymmetrical weakness. PUL had 16 pairs of items with highly correlated residuals (>0.3) (Table S2). One item from each pair was removed, and PSI remained stable at 0.86, indicating the dependency had not artificially inflated the PSI.

### Stability (differential item functioning [DIF]) and unidimensionality

The NSAD and PUL were examined for the presence of uniform and nonuniform DIF by age, sex, and genetic variant. When the NSAD was examined by age, five items demonstrated uniform DIF—rise from the floor, stand on heels, half kneel to stand (left and right), and stand on tiptoes. These items are more likely to be able to be performed in the younger and less affected cohort compared to the adult group. For sex, the only item with uniform DIF was stand on heels. For the genetic variant factor, nonunform DIF was present for the hop and stand on tiptoes items.

Uniform DIF was present for sex on PUL items shoulder flexion above shoulder height with 1 kg and supination. For age, uniform DIF was present for PUL items shoulder abduction arms above head, and both uniform and nonuniform DIF was present for tears paper and traces a path. No uniform DIF was present for genetic variant. Nonuniform DIF was present for push‐on light and tears paper.

## Discussion

Across a broad range of pediatric and adult patients with LGMDR9, we have determined a suitable battery of functional outcome measures, useful for both clinical management and as endpoints within clinical trials of investigational products. While there was no singular outcome that provided clinically meaningful measurement of function across all abilities and ages, the utility of each COA and PRO and its best context of use has been clarified in our study and may be useful for interpretation of current and future clinical trials enrolling patients with LGMDR9. A combination of COA, such as the 100 m, NSAD, and PUL, for example, provides the ability to measure a wide range of functional abilities to maximize clinical trial recruitment and inclusion. Conversely, a single test such as RFF would enable strict inclusion of a mildly affected homogeneous cohort, but results would be considered less generalizable to the larger population of patients with LGMDR9.

Findings from our sample confirm previous reports that on average patients with compound heterozygous variants exhibit earlier onset of symptoms and subsequent progression of disease compared to those who are homozygous for the common founder variant.^8^, ^9^ However, it is important to note that there is heterogeneity in individual patients and there are outliers in our cohort that do not follow this trend. Therefore, future research on the impact of genetic variants on disease progression will help to inform individual patient trajectories.

The psychometric properties and construction of ordinal scale assessments, the NSAD and PUL, were critically evaluated in our study. RMM confirmed the NSAD is a suitable and sensitive functional outcome measure of motor performance across ambulant and nonambulant individuals with LGMDR9. The NSAD demonstrated excellent test–retest reliability. Items of the scale fit well, with logical scoring categories, summed to a valid total score. Only one NSAD item (i.e., rolling) demonstrated disordered thresholds. This item was nearing ordered thresholds as the score of “1” rolling to the side was simply less frequently seen in this primarily ambulatory cohort; thus, this item was retained due to clinical relevance of the scoring order. Importantly NSAD can be used across disease progression, without a ceiling effect observed in this symptomatic cohort. Similarly, once upper extremity weakness presents, the PUL is a useful COA to quantify abilities sensitively across the span of disease progression. The scoring and inclusion of items characterize upper extremity function in this patient population and sum to a valid total score. While there is a ceiling effect in a stronger, less involved cohort, the PUL measures meaningful arm use across a wide range of abilities in nonambulatory patients without a measured floor effect. The relationship between upper limb function and NSAD demonstrates the need to objectively capture both for individuals with LGMDR9 with validated tools.

While the key aims of our study were to establish clinical trial readiness in LGMDR9 and to validate COA and PRO for research use, it is important to highlight findings from our study that can impact clinical care and proactive management of patients with LGMDR9. Use of these validated outcomes in standard clinical practice will enable clinicians to track patient progress and compare to expectations reported in published natural history studies. Similarly, as future disease‐modifying treatments are investigated and eventually approved, understanding the deviation from expected natural history will inform future care recommendations. Our study highlighted the current impact of LGMDR9 on function through the collection of a battery of PRO. The presence of pain is consistent across patients and should be proactively monitored and addressed. In addition, we identified thresholds for loss of key functional abilities leading to potential changes in independence and/or patient safety. For example, as a patient reaches threshold of 7 sec to complete the 10 m, the multidisciplinary care team should be discussing equipment or technology to support safe transfers in the event of a fall in the home or community. Nearing a 100 m time of 83 sec or an NSAD score of 35 points indicates patients may be having some difficulty with community ambulation. These thresholds can be useful to begin data‐driven conversations about part‐time assistive device use. The PUL Entry item should be considered a quick and useful clinical screening tool for classification of upper limb function as onset and progression of weakness is heterogeneous and may occur in patients who are ambulatory or nonambulatory. The distal domain items are particularly relevant for those with impaired upper limb function, beginning with those scoring at or below a 3 on the PUL entry (i.e., being able to take a cup with a 200 g weight to the mouth or less able).

Results from our multisite, international consortium completed a critical step in validating COA and PRO for use both clinically and in clinical trials in LGMDR9; however, there are limitations to our study that should be acknowledged. As our primary aim was to establish clinical trial readiness in this cohort, the enrollment criteria for our cohort were geared toward younger and ambulatory patients with LGMDR9. Stronger, less symptomatic patients (i.e., those taking <4 sec to complete the 10 m) were excluded from participation. Similarly, the cohort of patients who were transitionally ambulatory or nonambulant at baseline was restricted. Thus, our findings may not be fully generalizable to patients falling at the stronger or weaker ends of the spectrum of disease. In addition, while our consortium included international sites, most sites (i.e., 10 of 11) were based in the United States with our sole international site in Copenhagen, Denmark. Future work to expand enrollment to additional sites with better global representation will enhance the generalizability of findings to the full population of patients with LGMDR9. Data presented here are cross‐sectional in nature, and future evaluation of longitudinal changes in disease and sensitivity to COA and PRO in a prospective, longitudinal study is warranted and ongoing. Last, a better understanding of the utility of PRO used individually or in combination with functional COA will be important to truly achieving clinical trial readiness in LGMDR9.

## Conclusions

Our study highlights the critical need in COA and PRO development to ensure robust psychometric analysis of the data combined with expert physical therapist understanding of the impact of disease on motor function. We have developed and validated suitable COA to quantify function across a broad range of ages and abilities in patients with LGMDR9. Further work is to evaluate the utility of these scales in a prospective longitudinal study and ensure clinical meaningfulness with the patient community.

## Author Contributions

LNA, MKJ, JV, TM, KDM, LPL, MW, CCW, JMS, PBK, UD, and NEJ contributed to conception and design of the study. LNA, MKJ, KGP, KR, JV, RA, TM, AJ, AB, KML, SRHM, SKM, AMI, NFR, LP, LPL, TS, MW, SH, CCW, SS, MC, JMS, NS, MH, DGL, DJL, PBK, SH, UD, and NEJ contributed to acquisition and analysis of data. LNA and MKJ drafted a significant portion of the manuscript and figures. All authors contributed to review and approval of the final manuscript.

## Conflicts of Interest

Dr. Alfano received consultant fees from Asklepios Biopharmaceutical and ML Bio Solutions via ATOM International and received funding via institution and royalties from Sarepta Therapeutics. Dr. James receives consulting fees from ATOM International (covers consultancy services provided to Genethon, PTC Therapeutics, Sarepta Therapeutics) and consultant fees related to advisory boards via Newcastle University for Sarepta Therapeutics. Dr. Vissing received consultant fees for serving on advisory boards for Sarepta Therapeutics, ML Bio Solutions, and Atamyo. Dr. Mozaffar received consultant fees for serving in an advisory capacity for Ask Bio and research funding from ML Bio Solutions. Dr. Lowes receives funding via institution and royalties from Sarepta Therapeutics. Dr. Weihl received consultant fees for advising ML Bio Solutions and Sarepta Therapeutics. Dr. Kang received research support from ML Bio Solutions and Sarepta Therapeutics. Dr. Johnson received research funds from AskBio, ML Bio Solutions, and Sarepta Therapeutics. He has received consulting fees AskBio. Dr. Johnson has stock options in Repeat RNA Therapeutics, Angle Therapeutics, and Myogene Therapies. KGP, KR, RA, AJ, AB, KML, SRHM, KDM, MAI, NFR, LP, TS, MW, SH, SS, MC, JMS, NS, MH, DGL, DJL, PBK, SH, UD have nothing to report.

## Supporting information


Data S1.

## Data Availability

The data that support the findings of this study are available upon request from the corresponding author. The data are not publicly available due to privacy or ethical restrictions.
