# Supplementary material for: Prospective observational study of FKRP‐related limb‐girdle muscular dystrophy R9: A GRASP consortium study
Source: Ann Clin Transl Neurol. 2024 Dec 15;12(2):332–44. doi: 10.1002/acn3.52276 (PMC11822816; doi:10.1002/acn3.52276)
Supplement: Supplementary file 1 — Data S1. [file ACN3-12-332-s001.docx]

Supplemental Fig 1: Person-item threshold distribution of North Star Assessment for limb girdle-type dystrophies (NSAD) completed across baseline visits demonstrates good match and spread of items across the cohort.


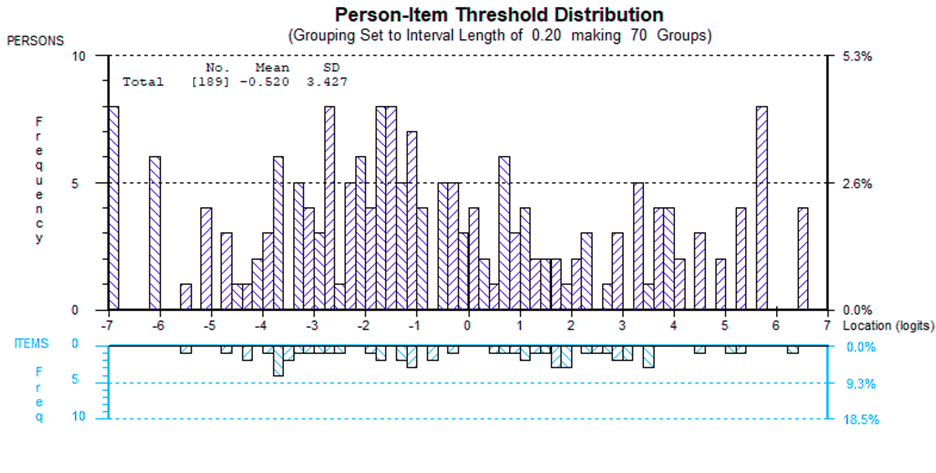


Supplemental Fig 2: NSAD item demonstrate 28 of 29 ordered scoring categories, depicted here in order from least to most difficult. Though most patients are able or unable to roll from supine to prone, this item is approaching ordered thresholds for the partial roll indicating the 3-point scoring is meaningful in this population and is an approximation although not identical to rolling in bed at home.


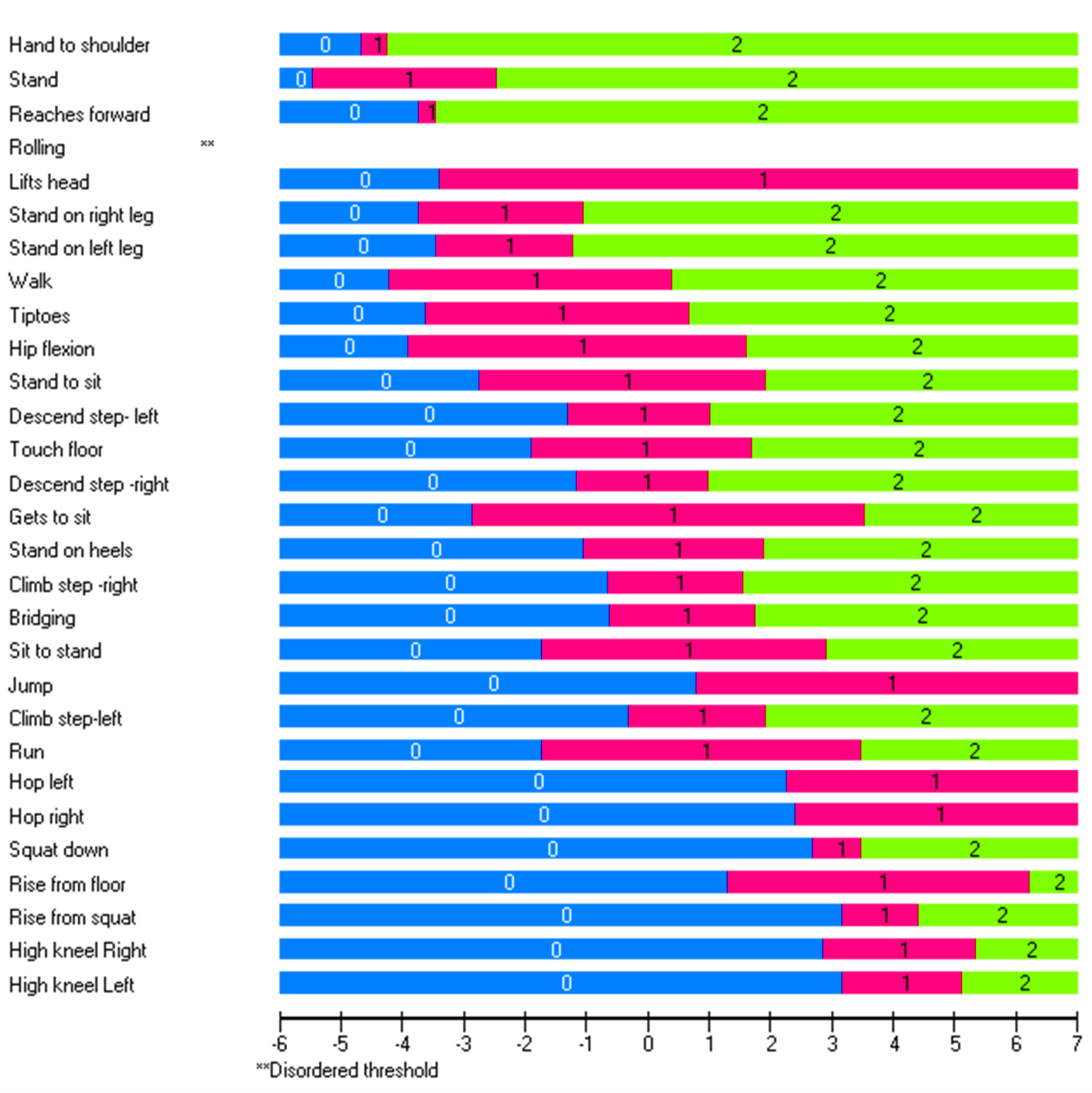


Supplemental Fig 3: A) Person-item threshold distribution of Performance of Upper Limb 2.0 (PUL) completed across baseline visits demonstrates good match and spread of items across the cohort. A ceiling effect is noted in this primarily stronger, ambulatory cohort. B) Person-item threshold distribution separated by ambulatory group (blue = ambulant; red = non-ambulant at baseline)


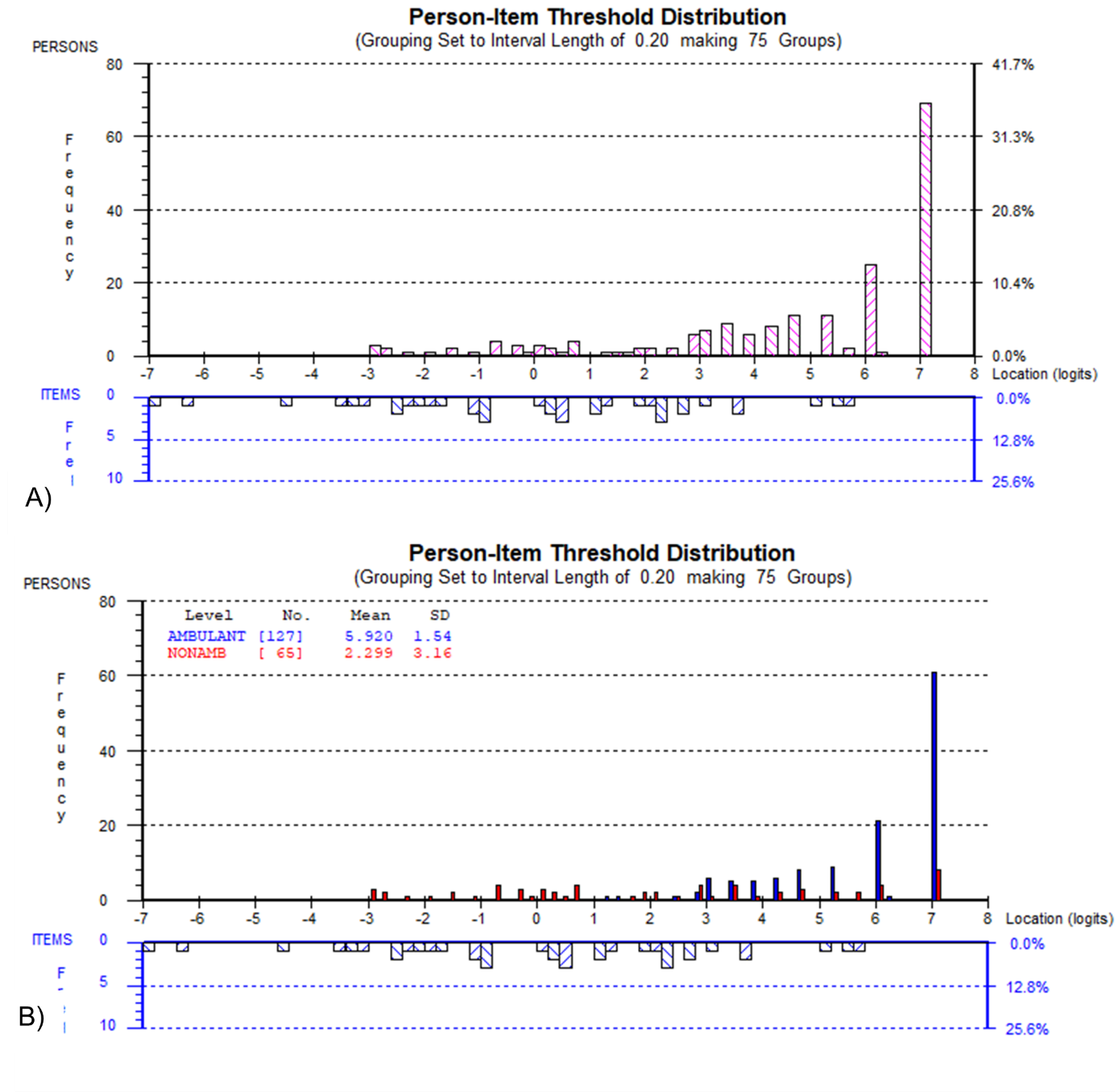


Supplemental Fig 4: PUL items demonstrate 21 of 22 ordered scoring categories, depicted here in order from least to most difficult. The singular disordered item, raising arms to shoulder height, demonstrates close to ordered category probability curves indicating the 3-point scoring is likely still meaningful in this population.


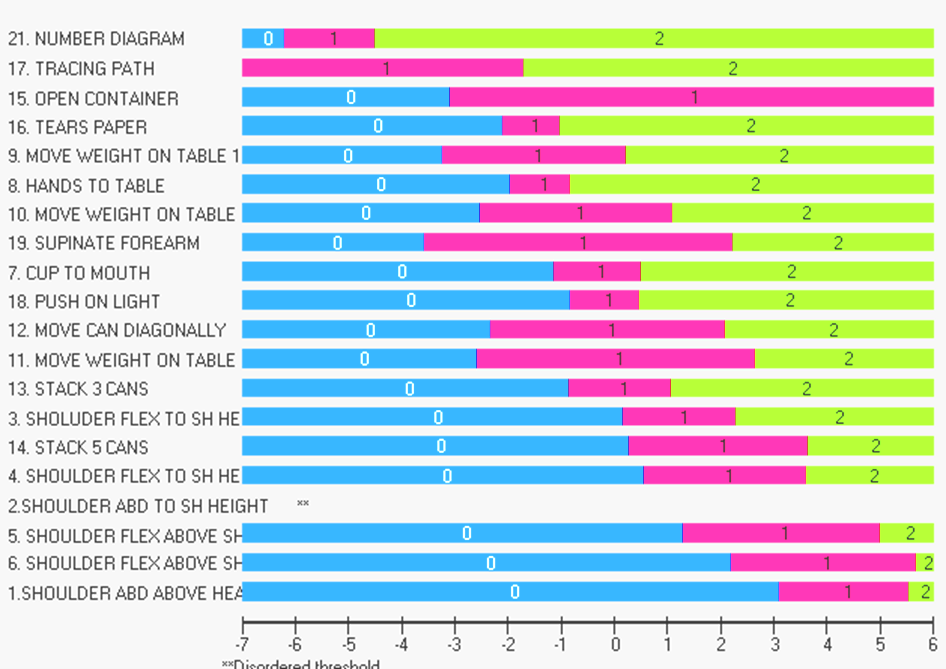


Supplemental Table 1: Individual item fit for the NSAD 29 items in threshold location: order of difficulty, easiest to most difficult.

| **Item** | **Item number** | **Item location** | **Standard error** | **Fit Residual** | **χ2** | **χ2 p values** |
| --- | --- | --- | --- | --- | --- | --- |
| Hand to opposite shoulder | 2 | -4.456 | 0.212 | -0.106 | 1.437 | 0.488 |
| Stand | 10 | -3.952 | 0.194 | 0.085 | 2.248 | 0.325 |
| Reaches forward | 7 | -3.573 | 0.18 | 0.713 | 112.348 | 0.000 |
| Rolling supine to prone | 5 | -3.42 | 0.185 | -0.039 | 2.93 | 0.231 |
| Lifts head | 1 | -3.376 | 0.25 | 0.582 | 23.64 | 0.000 |
| Stand on one leg- right | 12 | -2.382 | 0.169 | 0.95 | 0.995 | 0.608 |
| Stand on one leg - left | 13 | -2.322 | 0.165 | -0.519 | 1.634 | 0.442 |
| Walk | 11 | -1.9 | 0.185 | 0.086 | 5.258 | 0.072 |
| Stand on tiptoes | 28 | -1.461 | 0.18 | 7.184 | 43.309 | 0.000 |
| Hip flexion in supine | 3 | -1.127 | 0.191 | -0.15 | 1.36 | 0.506 |
| Stand to sit | 9 | -0.388 | 0.18 | -2.029 | 3.412 | 0.182 |
| Descend box step -left | 17 | -0.128 | 0.167 | -0.508 | 9.321 | 0.009 |
| Touches floor from standing | 18 | -0.094 | 0.172 | -1.744 | 2.516 | 0.28 |
| Descend box step - right | 15 | -0.074 | 0.167 | -0.308 | 4.955 | 0.0840 |
| Gets to sitting | 6 | 0.354 | 0.188 | -1.989 | 2.4 | 0.301 |
| Stand on heels | 20 | 0.444 | 0.174 | 1.812 | 22.033 | 0.000 |
| Climb box step -right | 14 | 0.475 | 0.173 | -2.23 | 6.211 | 0.045 |
| Bridging in supine | 4 | 0.581 | 0.175 | -0.032 | 3.827 | 0.148 |
| Sit to stand | 8 | 0.604 | 0.178 | -3.188 | 5.336 | 0.069 |
| Jump | 21 | 0.81 | 0.252 | -1.672 | 15.799 | 0.000 |
| Climb box step - left | 16 | 0.819 | 0.178 | -2.284 | 5.676 | 0.058 |
| Walk/run 10m | 29 | 0.889 | 0.18 | -0.955 | 0.613 | 0.736 |
| Hop left leg | 23 | 2.274 | 0.286 | -1.193 | 5.046 | 0.080 |
| Hop right leg | 22 | 2.432 | 0.29 | -1.069 | 3.67 | 0.159 |
| Squat down | 24 | 3.102 | 0.214 | 0.168 | 0.653 | 0.721 |
| Rise from floor | 19 | 3.784 | 0.223 | -1.223 | 3.042 | 0.218 |
| Rise from squat | 25 | 3.804 | 0.222 | -0.675 | 2.725 | 0.256 |
| High kneel to stand right | 26 | 4.125 | 0.229 | -0.928 | 1.557 | 0.459 |
| High kneel to stand left | 27 | 4.155 | 0.229 | -0.987 | 2.756 | 0.252 |

^a^ Items that ‘misfit’ the overall scale (items should lie in range of SD +/- 2.5).

Supplemental Table 2: Individual item fit for the PUL 2.0* items in serial location:

| **Item** | **Item number** | **Item location** | **Standard error** | **Fit Residual** | **χ2** | **χ2 p values** |
| --- | --- | --- | --- | --- | --- | --- |
| Number diagram | 21 | -5.351 | 0.876 | -0.16 | 0.136 | 0.934 |
| Traces path | 17 | -4.346 | 0.443 | 0.514 | 24.441 | 0.000 |
| Open container | 15 | -3.09 | 0.584 | 0.223 | 0.481 | 0.786 |
| Tears paper | 16 | -1.554 | 0.31 | 1.641 | 104.613 | 0.000 |
| Move weight on table 100g | 9 | -1.49 | 0.308 | -1.334 | 1.978 | 0.372 |
| Hands to table | 8 | -1.391 | 0.303 | -0.62 | 0.953 | 0.621 |
| Move weight on table 500g | 10 | -0.707 | 0.28 | -2.406 | 5.199 | 0.074 |
| Supination | 19 | -0.658 | 0.267 | 4.016 | 63.344 | 0.000 |
| Cup to mouth | 7 | -0.304 | 0.266 | -1.996 | 2.509 | 0.285 |
| Push on light | 18 | -0.171 | 0.262 | 1.263 | 80.782 | 0.000 |
| Move can diagonally | 12 | -0.116 | 0.256 | -1.932 | 2.654 | 0.265 |
| Move weight on table 1kg | 11 | 0.038 | 0.246 | -2.191 | 4.216 | 0.121 |
| Stack three cans | 13 | 0.119 | 0.256 | -2.398 | 4.784 | 0.091 |
| Shoulder flexion to shoulder height | 3 | 1.241 | 0.227 | -3.069 | 5.088 | 0.078 |
| Stack five cans | 14 | 1.982 | 0.207 | -3.743 | 5.006 | 0.082 |
| Shoulder flexion to sh height 500g | 4 | 2.091 | 0.206 | -3.527 | 6.92 | 0.031 |
| Shoulder abduction to shoulder height | 2 | 2.261 | 0.212 | 0.114 | 4.341 | 0.114 |
| Shoulder flex above sh height 500g | 5 | 3.159 | 0.197 | -3.565 | 16.39 | 0.000 |
| Shoulder flex above sh height 1kg | 6 | 3.947 | 0.195 | -1.743 | 7.886 | 0.019 |
| Shoulder abduction above head | 1 | 4.339 | 0.182 | -0.248 | 11.76 | 0.003 |

* Two items removed by RMM as all cohort achieved full scores- pick up 10g weight and pick up coins
